# The feasibility of training nnU-Net to segment pediatric Wilms tumor with small datasets

**DOI:** 10.3389/fonc.2026.1841994

**Published:** 2026-10-22

**Authors:** M.A.D. Buser, R.F. Meijer, A.F.W. van der Steeg, M.H.W.A. Wijnen, M. M. van den Heuvel-Eibrink, A.S. Littooij, G. De Donno, A. De Luca

**Affiliations:** 1 Princess Máxima Center for Pediatric Oncology, Utrecht, Netherlands; 2 Division of Childhealth, Wilhelmina Childrens’ Hospital, Utrecht, Netherlands; 3 Department of Radiology and Nuclear Medicine, University Medical Center Utrecht, Utrecht, Netherlands; 4 Image Sciences Institute, University Medical Center Utrecht, Utrecht University, Utrecht, Netherlands

**Keywords:** abdominal oncology, deep learning, MRI, segmentation, Wilms tumor

## Abstract

**Introduction:**

Segmentation of magnetic resonance imaging (MRI) can be used in the clinical care of patients with (suspected) Wilms tumor (WT). Manual segmentation, however, is time consuming and user dependent. Deep learning can be used to automate this, but is often associated with the need for large training datasets to be trained successfully, which is inherently challenging in pediatric populations. The aim of this study was to determine the effect of the number of training patients on automated WT segmentation.

**Methods:**

56 patients with a suspected WT were included in this retrospective study. The pre-operative MRI scans were divided in a training (n = 45) and test (n = 11) set. The segmentation was based on nnU-Net, an out-of-the-box framework for image segmentation. nnU-Net was trained on a random selection of five patients, and the resulting segmentation method was tested on a fixed test set of 11 patients. Next, patients were added to the training dataset with incremental steps of five patients, up to the full dataset of 45 patients. This process was repeated 5 times to account for random sampling. The Dice score was used as measure for segmentation performance. We pragmatically defined a Dice score of > 0.80 as a reference level for segmentation performance.

**Results:**

The lowest median Dice score was obtained when using a subset of five training patients (0.38). The highest median Dice score was observed for training on 45 patients (0.91). An median Dice score of 0.80 was reached using a minimal subset of 10 training patients. The Dice score showed dependency on the random selection of the training patients, with the largest spread in performance observed in the training set of five patients. A large spread in performance between the 11 test set patients was observed.

**Discussion:**

Using this specific training and test cohort, a median Dice score of 0.80 was reached with just 10 training patients, however, the results showed considerable variation in the individual test patients. The performance spread between the random subsampling of the training patients highlights the importance of clinically representable datasets when dealing with the small dataset sizes inherent to pediatric oncology.

## 1 Introduction

Magnetic resonance imaging (MRI) is the imaging modality of choice in children with renal tumors, due to the absence of radiation exposure and the excellent soft tissue contrast (1). Delineation of the tumor on MRI (e.g. segmentation) is an addition to the diagnostic and response assessment workflow. It was used successfully in pediatric abdominal tumor patients, for example for volume response measurements and for the creation of 3D models to support surgical planning. This is currently primarily performed through manual segmentation (2–4). However, the process of manually segmenting MRI scans is time consuming and user dependent (3, 5). Automating the segmentation process minimizes these limitations, making clinical implementation of MRI segmentation more feasible.

Deep learning (DL) approaches have become the standard to automate tumor segmentations in adult oncology (6). A key challenge in applying deep learning to pediatric tumor segmentation is the scarcity of training data, inherent to the rarity of these conditions. A recent summary of the literature revealed that in practice there is a large variation in the dataset size used to train DL applications, with examples of successful segmentation with as little as 28 scans from 17 patients (7). While several of the articles included in the review showed promising segmentation results, it remains difficult to reach a conclusion about the amount of training data needed to create a successful segmentation method for each pediatric tumor group. The scarcity of data might limit the translation from DL-based segmentation methods developed in adult, data-rich populations.

The current state-of-the-art for image segmentation is nnU-Net (8), which has shown promising results in both adult and pediatric populations (9, 10). Due to the extensive algorithm optimisation and integrated data augmentation, nnU-Net might not need extensive amounts of data to create an accurate segmentation result. This, however, remains untested in segmentation of tumors in the pediatric population. In this study, we investigate the trade-off between training dataset size and segmentation accuracy of nnU-Net, with a proof-of-concept in children with suspected Wilms tumors (WTs). Previous results (2) have shown the feasibility of achieving excellent automated segmentations on this tumor provided a relatively large training dataset. In this study, we evaluate the feasibility of training such a model on lower number of patients, providing insights that might generalize to many smaller centers interested in training DL methods for WTs.

## 2 Methods

### 2.1 Patients

A cohort of pediatric patients with renal tumors, suspected to be a WT, diagnosed and treated in the national center (the Princess Máxima Center for Pediatric Oncology, Utrecht, The Netherlands) between July 2018 and July 2024 was included in this study. This study was approved by the Medical Ethical Board within the informed consent for treatment protocol (METC 18-033/M) (11). Patients were excluded when imaging was incomplete (n = 4) or when no informed consent for the SIOP-RTSG-2016 UMBRELLA study was present (n = 1). The study included patients with tumors confined to a single kidney (unilateral), as well as those with involvement of both kidneys (bilateral), yielding a total of 56 patients and 64 tumors. Following the SIOP-RTSG 2001 or the SIOP-RTSG 2016 UMBRELLA protocol, most patients were treated with pre-operative chemotherapy unless age or other factors contraindicated this (11–13).

### 2.2 Magnetic resonance imaging

MRI scans were performed at the time of diagnosis and prior to surgery, following the SIOP-RTSG 2016-UMBRELLA protocol. Only imaging before resection was included, as this is in line with the intended aim of the creation of 3D models for surgical planning.

The MRI scans were acquired using a 1.5T MRI system (Ingenia; Philips Medical Systems, Best, The Netherlands) and included a 3D T_2_-weighted sequence alongside fat-suppressed T_1_-weighted imaging with and without intravenous contrast (Gadovist, Bayer Pharma, Berlin, Germany, 0.1 mmol/kg body weight) (**Table 1**).

**Table 1:** MRI parameters.

Parameters	T_1_-weighted with fat suppression	3D T_2_-weighted
Sequence type	Gradient Echo	Turbo Spin Echo
Repetition time (ms)	5.5	459
Echo time (ms)	2.7	90
Flip angle	10°	90°
Slice thickness (mm)	3.0	1.15
Voxel spacing (mm^2^)	0.74 × 0.74	0.83 × 0.83

### 2.3 Manual segmentation

Tumor segmentations were created on the pre-operative T_2_-weighted images by one of three independent observers (technical physicians) in the software package 3D Slicer (14). The images were randomly distributed between the three observers and a standardized segmentation protocol, as described in Buser et al. (2022) was followed. If needed, the T_1_-weighted images were consulted to better define tumor boundaries. The segmentations were all reviewed by an experienced radiologist (A.S.L.) and corrected by the first author if necessary.

### 2.4 Segmentation models

Automated segmentation models were created using nnU-Net (v2.0.1), a self-adapting framework for biomedical image segmentation (8). T_2_-weighted images together with binary segmentation masks of the tumor were used as input to the “3d_fullres version” of nnU-Net. The models were trained for 500 training cycles (epochs). No additional pre-or postprocessing was applied. No random seed was manually specified. The rest of the parameters were set to nnU-Net defaults.

To create the training and test dataset, an 80% and 20% split was used. First, unilateral patients were randomly assigned to the training and test dataset. Next, bilateral patients were divided separately between the training (n = 6) and test (n = 2) datasets to ensure that both contained a representative distribution of such cases. This was necessary because bilateral cases involve both kidneys of the same patient, making this group unsuitable to be split together with the unilateral patients. Together, this resulted in 45 patients for training (the full training dataset) and 11 for testing (the test dataset) (**Figure 1**). To assess the comparability of the training and test set, these datasets were statistically compared on both a patient and tumor level. First, sex, age at the time of imaging and the presence or absence of pre-operative chemotherapy were compared using the Mann–Whitney test and Fishers exact tests for all patients. Secondly, the split for unilateral patients was tested based on tumor volume (Mann-Whitney U test) and the SIOP-RTSG histological classification (Chi-squared test). Because the segmentation models process the entire patient image rather than individual kidneys, testing the data split on tumor-level characteristics in bilateral cases was not meaningful and therefore not performed. As no statistically significant differences were observed between the training and test cohort for the evaluated characteristics, no resplit was necessary.

**Figure 1:**
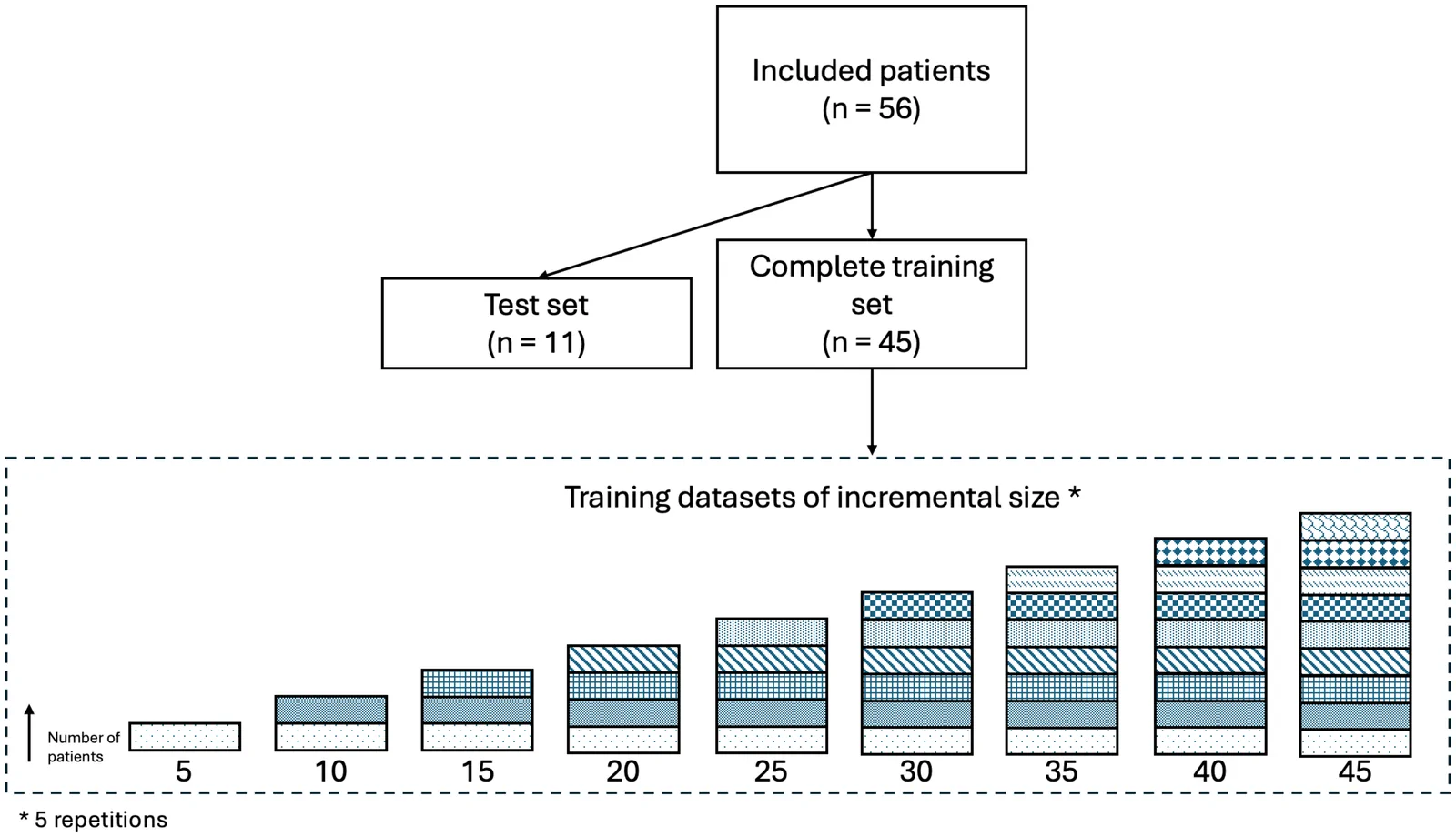
The overview of the study set-up. In total, 56 patients were included, of which 11 were used as test dataset and the other 45 for the full training dataset. From the full training set, incremental patients were added in steps from 5 to 45 to form subsets and used to train a nnU-Net model. The whole process was repeated 5 times to account for the effect of random sampling.

To evaluate the effect of the number of training patients, each individual nnU-Net was trained by iteratively increasing the training subset. To initialize this, 5 patients were randomly selected from the full training set and the nnU-Net was trained solely on these 5 patients (**Figure 1**). This was repeated by adding 5 additional patients, up to a maximum of 45 patients to nested incremental sets of training data and each nnU-Net training was repeated from scratch. This resulted in a total of 9 segmentation models, trained on nested, incremental sets of data. The iterative training procedure was repeated 5 times (a pragmatic compromise between characterizing sampling variability and maintaining a feasible computational load) to evaluate the impact of different random selections of patients, resulting in 45 trained nnU-Net models.

### 2.5 Performance evaluation

Each segmentation model was tested on a fixed test set of 11 patients. The test set was fixed to ensure the comparability of the results obtained with different training sets. The segmentation performance was evaluated using the Dice similarity coefficient (Dice score), as is common in the field (15). The Dice score was calculated on a per-patient level, with no separate evaluation in case of a patient with a bilateral tumor.

To visualize the results, the median performance was calculated across test patients. Subsequentially, the median, 25^th^ and 75^th^ interquartile were determined between the 5 different training repetitions and plotted as a function of the number of patients in the training subset. For the median Dice performance per patient, the interquartile range was not plotted for clarity of the plot. To judge the potential for clinical applicability, two thresholds for the Dice performance were chosen. First, a Dice score of 0.00 representing total segmentation failure. Second, a Dice score of 0.80 as a reference level, in line with the variation between observers in the segmentation of Wilms tumors (5). A performance plateau was defined as a Dice performance gain smaller than 0.01 between one enlargement of training dataset size.

## 3 Results

### 3.1 Patients

The median age of the included patients (n = 56) at time of scanning was 37.5 months (min – max: 8 – 110). The majority of the patients had a unilateral, left sided tumor (n = 29), while 7 patients with bilateral tumors were included. The patient characteristics are shown in **Table 2**. Most patients were pretreated with chemotherapy, except when younger than six months (n = 6), according to the current SIOP-RTSG Umbrella protocol. Mixed (n = 14) or regressive type tumors (n = 15) were most prevalent (**Table 3**). The median tumor volume was 324 ml (min – max: 0.58–1792 ml).

**Table 2:** Characteristics of the included patients.

Characteristics	Category	Value (n = 56)
Age at scanning moment in months, median (min-max)		37.5 (8 – 110)
Sex, n (%)	Male	27 (48)
	Female	29 (52)
Tumor site, n (%)	Left	29 (52)
	Right	20 (36)
	Bilateral	7 (12)
Pre-treated with chemotherapy, n (%)	Yes	50 (89)
	No	6 (11)

**Table 3:** Tumor characteristics for the unilateral tumors.

Characteristic	Category	Value (n = 48)
Histological diagnosis, n (%)	Regressive	15 (31)
	Mixed	14 (28)
	Stromal	7 (14)
	Diffuse anaplastic	4 (8)
	Completely necrotic	2 (4)
	Epithelial	2 (4)
	Nephrogenic rest	2 (4)
	Cystic nephroma	1 (2)
	Benign kidney tumor	1 (2)
Tumor volume [ml], median (range)	-	324 (0.58 – 1792)

The training and test set showed no significant differences for gender (*p* = 0.096) or age (median 36.00 months, 41.00 months, *p* = 0.55). Pathological diagnosis was also balanced between groups (*p* = 0.45). Furthermore, no significant differences were observed between the training and test sets with respect to tumor volume (mean 488 ml, 452 ml, *p* = 0.60). Pretreatment was also similarly distributed between the two groups: with 41/45 (91%) of patients in the training set and 9/11 (82%) in the test set having received chemotherapy (*p* = 0.59).

### 3.2 Segmentation performance

In **Figure 2**, the relationship between the Dice score and the size of the training subset is plotted. The lowest median Dice score was 0.38 for 5 training patients, and the highest median Dice score was 0.91 for 45 training patients. The interquartile range for 5 training patients was the largest (0.28 – 0.61), while for 45 training patients it was the smallest (0.91 – 0.92). The highest improvement was observed between 5 and 10 training patients: from a median Dice of 0.38 to 0.84. A plateau in training performance was not reached before reaching the full training set of 45 patients, as a limited increase of the Dice score between the subset of 40 and 45 training patients was still present (from 0.89 to 0.91).

**Figure 2:**
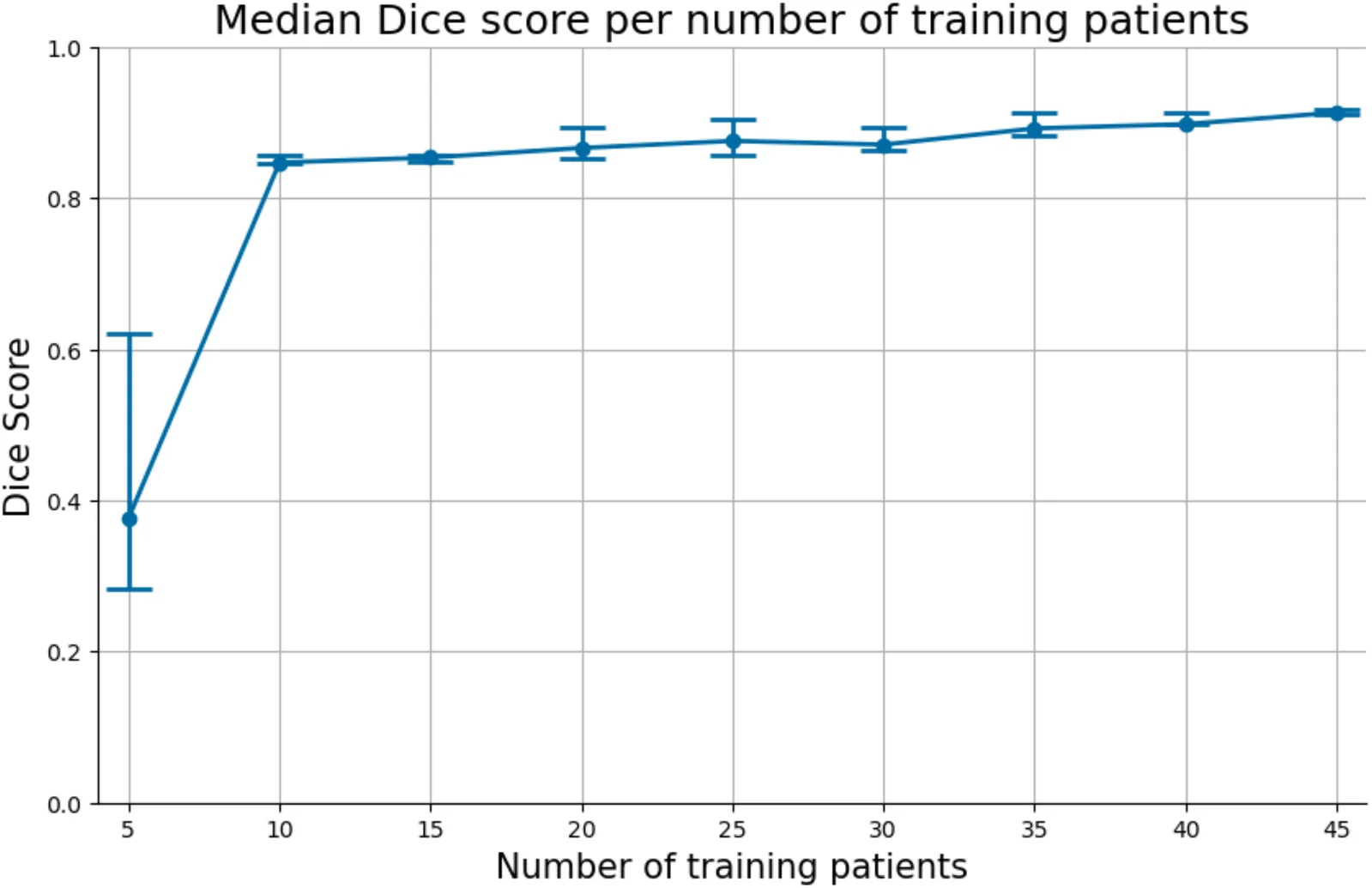
The median Dice performance on the test dataset as a function of the number of patients in the training subset. The error bars indicate the interquartile range for the 5 different training repetitions. The biggest increase in performance can be observed between 5 and 10 training patients. No plateau was reached for the median Dice score, as a limited increase in Dice score can still be observed between the subsets of 40 and 45 patients.

In **Figure 3**, the median performance for each of the 11 patients in the test set is depicted. The highest Dice score was reached by patient 5, with a median Dice score of 0.98 for the nnU-Net models trained on a subset of 35, 40 and 45 patients. The highest initial Dice score was reached by patient 2 (Dice = 0.85). The only patient that never reached the threshold Dice > 0.00 was patient 8. From the rest of the 10 test patients, the lowest Dice score was observed in patient 7, with a median Dice score of 0.65 for the nnU-Net models trained on 45 patients.

**Figure 3:**
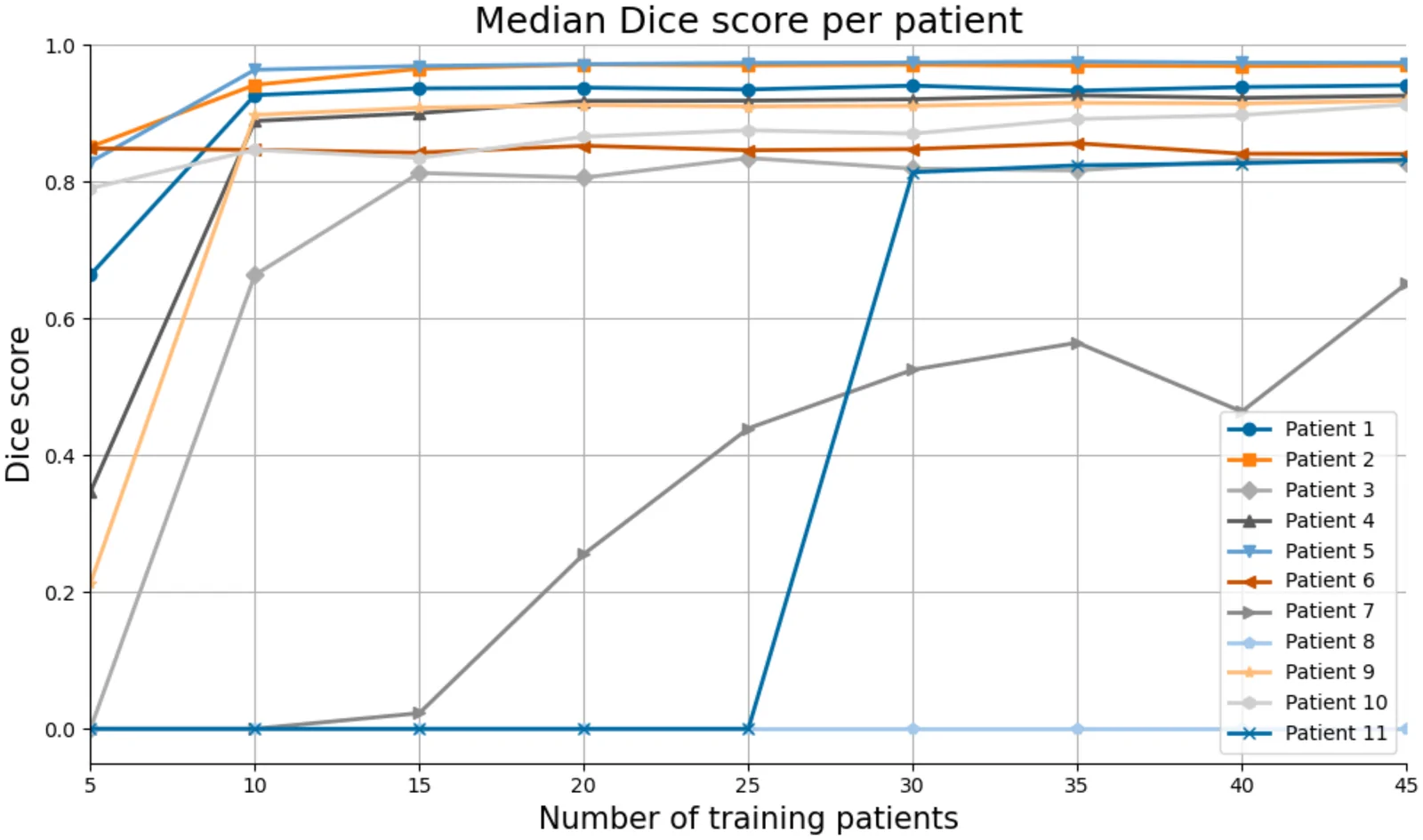
The median Dice performance between each training repetition on each test patient as a function of the number of patients in the training set. Note that patient 8 never reached a non-zero Dice. The performance with the largest increase was for patient 11. The other patients show the largest performance increase between 5 and 10 training patients.

The proportion of patients with a Dice score > 0 differed among training repetitions, but all reached a maximum of 91% (10 out of 11 patients). Training repetition 4 reached this maximum with the least number of training patients (15). This contrasted with training repetition 1 only reaching the maximum after 35 patients (**Figure 4A**). The percentage of patients who reached the reference level Dice score (Dice > 0.80) got to a maximum of 81% (9 of 11) for all of the training repetitions. The number of training patients required to reach this maximum ranged from 30 patients to 45 patients (**Figure 4B**).

**Figure 4:**
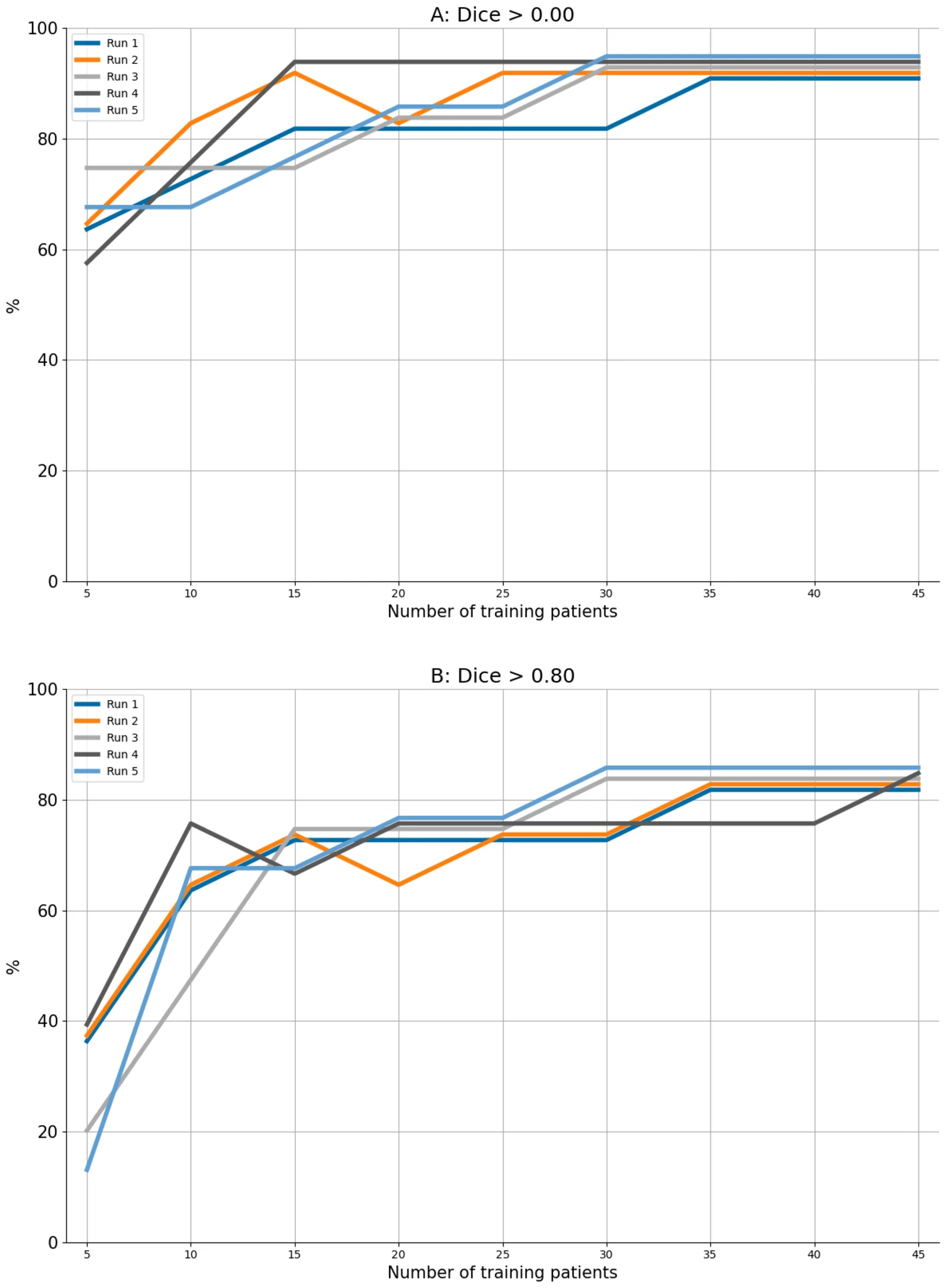
Segmentation rates across test patients as a function of the number of patients in the training set for each of the training repetitions. Subfigure **(A)** patients with Dice > 0.00 across number of training patients. Subfigure **(B)** The rate of patients who reached the reference level Dice score (Dice > 0.80). Please note that if lines were completely on top of each other, a small offset was used for visualisation purposes.

### 3.3 Failure analysis

In **Figure 5**, the segmentation results of four different patients are depicted for the nnU-Net models with 5, 25 and 45 training patients. The patient with the lowest overall increase in Dice score, patient 2, has a large, clearly defined tumor. The segmentation trained on 5 patients mostly includes the hyperintense parts, but subsequent segmentations do include the whole tumor. Next, patient 11 required the largest amount of training patients to get a Dice score > 0.80. This patient has a completely necrotic tumor, which is small, midpolar and displays an intensity comparable to the surrounding kidney parenchyma. Patient 7 (with a mixed type of WT) shows a general trend of an increasing segmentation performance but never reaches performance stability nor a Dice score of > 0.80. This tumor is small and cystic. Patient 8, a patient with bilateral lesions (of regressive type) in the lower pole of both kidneys, never showed any segmentation result. The clinical data of the worst performing patients is depicted in **Table 4**. Noticeably, all these patients had a small tumor (<13 ml) compared to the median tumor size in the whole dataset (>300 ml).

**Figure 5:**
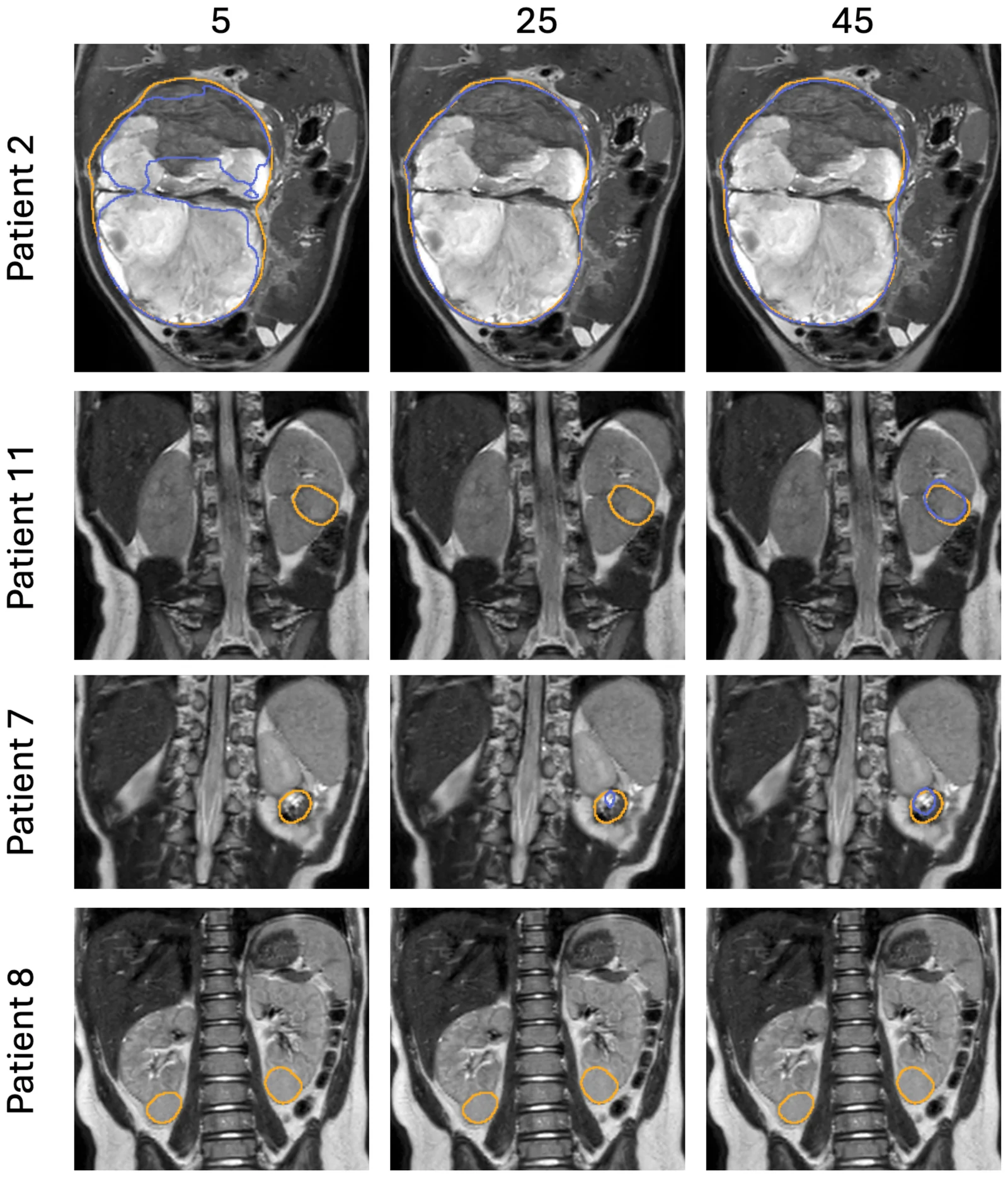
The segmentation results on the pre-operative T_2_-weighted scan for four test patients for different amounts of training data, as indicated in the title of each column. In orange the outline of the ground truth (manual segmentation) and in blue the automated segmentation result. The top row: segmentations of patient 2, which showed the highest initial Dice score (stromal WT). Second row: example segmentations of patient 11 (completely necrotic), which required the highest amount of training patients to reach the reference level Dice score (> 0.80). Third row: example segmentations of patient 7 (mixed type WT), which never reached the reference level (Dice > 0.80). Bottom row: example segmentations of patient 8 (regressive), which never got a Dice > 0.00.

**Table 4:** Overview of patients with the worst performance.

Example	Maximum dice	Volume (ml)	Type (percentage of total)
Patient 7	0.68	3.4	Mixed (28%)
Patient 8	0.00	7.02	Regressive (31%)
	0,00	8.03	Regressive (31%)
Patient 11	0.84	12.06	Completely necrotic (4%)

The reported Dice scores are of run 1.

## 4 Discussion

We investigated the relation between the training dataset size and segmentation performance for suspected pediatric Wilms tumors on MRI. This is particularly relevant as small datasets are an inherent problem in pediatric oncology research. Although the median segmentation performance was highest while training on the full training set (Dice = 0.91), the highest improvement was observed between 5 and 10 training patients. For this specific dataset split, a reference level Dice score of 0.80 or higher was reached for most of the test set patients using a training subset of as little as 10 training patients.

We focused on nnU-Net segmentation, as it can be readily applied in different settings out-of-the-box, without the need for extensive optimalisation efforts (8). In a previous study, we showed that nnU-Net segmentation for WT performed with a median Dice score of 0.90, using 45 patients in total in a 5-fold cross validation (with 36 training patients per fold) (2). Our current research did not show a substantial increase in maximum Dice, despite increasing the overall size of the previous, overlapping dataset. However, an implied training plateau could not be observed in our set-up, likely due to the small overall size of the training dataset. While in our experiments improvements became marginal with larger training sizes, further improvements of the segmentation results might be possible with larger datasets. However, Müller et al. showed an inter-observer Dice agreement of around 0.80 for pre-operative Wilms tumor segmentation. This provides a useful context for interpreting further improvements in Dice scores, as variability between human observers may limit the achievable overlap. Still, it is important to note that the Dice score is an important metric during the development and (retrospective) validation of segmentation methods, but it should not be considered a direct indication for clinical usability without clinical validation studies (16).

For the number of training patients of 5, the IQR between the training repetitions was 0.28 – 0.61. At 10 training patients, the IQR was 0.85 – 0.86, suggesting a rapid decrease in variability with the addition of training patients. The IQR for 45 patients was 0.91 – 0.92. This high variability between training repetitions suggests that when dealing with such limited amounts of training patients, it is important to have a set of patients that adequately represents the general population of patients with the disease. Therefore, it is important to further investigate the effect of patient- or disease specific factors, together with the presentation of the tumor on MRI, on the dataset-level segmentation performance. This might even lead to exclusion of patients with a less common presentation.

We did not repeat the sampling of the test set to allow consistent evaluation between experiments, even though the selection of the test patients also influences the results. While the training and test sets showed no statistically significant differences in the evaluated characteristics, it cannot be established if the test set is representative of the broader patient population due to the small overall size (n = 11) of the test set. Importantly, we included all patients with a WT, regardless of size of the tumor, number of tumors per kidney and patients with bilateral disease. Due to this, large performance differences were observed between individual test set patients. Most patients showed similar segmentation curves, with a large performance increase between training on 5 patients and 10 patients, but a relatively small increase thereafter. However, in three cases, where all tumors were small and had an intensity comparable to the kidney parenchyma, a limited performance was observed, sometimes even including the absence of any segmentation. This shows that the spread in segmentation results is patient specific, even when increasing the training dataset size. In small datasets, particularly in rare diseases with high variability between patients, attempting to include every possible rare representation might not necessarily result in a more robust model. Given the limited number of available patients, distributing the training data across a broad range of uncommon presentations might also reduce the number of examples for more common patients. Moreover, it is difficult to design a dataset that is fully representative of all possible clinical presentations, as unexpected, uncommon cases will most likely still occur. For example, in our prospective evaluation of WT segmentation we included a patient with an ureteral Wilms tumor, a rare presentation that was not considered during model development (16). Therefore, it might be preferable to develop an algorithm that provides robust performance within a clearly defined clinical scope, while identifying situations in which lower performance is expected. As the intended use of such algorithms will likely involve a “human-in-the-loop” workflow, identifying cases in which automated segmentation may be unreliable could help define an appropriate scope of use and ensure that these cases receive appropriate human review.

It is important to note that while patients with a suspected Wilms tumor were included, five patients had nephroblastomatosis (all in the training set), an unconfirmed tumor type (in the test set) or a non-WT tumor type (in the training set). As pediatric patients generally do not receive a biopsy before surgery, the inclusion of non-WT patients and patients with nephroblastomatosis is in line with the clinical practice in the SIOP-RTSG setting (11). Although nephrogenic rests can mimic small WT, this might have influenced the segmentation results (17).

The literature regarding the required dataset size for the segmentation of WT is limited. A recent study by Ding et al. investigated the effect of training set size in segmentation of organs at risk (OAR) in computed tomography scans of pediatric patients receiving radiotherapy for an abdominal tumor (including WT) (18). However, in this particular study, tumor segmentation was not the research focus. They found a median Dice score of 0.85 across all OARs for as little as ten training patients, with little improvement in a training dataset size larger than 50 patients. Although their imaging technique and segmentation target makes a comparison with our study not possible, this is a promising finding. The study by Gottlich et al. also provides interesting contextualisation, as they performed the only adult study focusing on patients with a kidney tumor, in this case renal cell carcinoma (RCC) (19). They investigated both CT and MRI segmentation (T_2_-weighted) and showed that for MRI, 389 patients were needed for a stable nnU-Net performance, resulting in a maximum of a Dice score of 0.92. However, they did not include training sizes smaller than 50 patients and focused only on RCC, preventing direct comparison with our results in patients with a WT.

We performed this study on pre-operative scans of patients with a suspected WT, as this falls within our intended use of surgical planning. As the included patients were treated according to the SIOP-RTSG-2016 UMBRELLA protocol, this means most cases in the dataset were pretreated tumors. Muller et al. showed there was only a limited difference between Wilms tumors at presentation and after pre-operative chemotherapy for both manual and automated segmentation, showing a potential in extending the dataset size by adding diagnostic scans of patients (5).

While the study was intentionally designed around our clinical workflow, a number of choices and constraints should be considered when interpreting the results. Our segmentation approach was trained and applied only to T_2_-weighted scans, as this is in line within our current protocol of creating pre-surgical 3D models. Previous research showed no overall segmentation improvement when using T_1_-weighted imaging with or without contrast (20). However, on an individual patient level, the use of multiple contrasts might improve the segmentation performance, especially in the smaller and less defined tumors. Although segmentation on anatomical sequences is more common, segmentation of pediatric oncology on microstructural MRI sequences such as diffusion weighed imaging (DWI) is gaining interest, where segmentation can be used to better quantify tumor properties and treatment effects (21).

We used imaging of patients with WT, a relatively common tumor with excellent segmentation results due to the well-defined and predominantly spherical shape (2, 5). Other tumor types, such as neuroblastoma, with a more extensive pre-operative treatment and invasive growth pattern (9), are expected to be more challenging to segment. Moreover, research on the segmentation of relatively common tumor types, such as WT and neuroblastoma, has been more extensive than research on less prevalent tumor types (7, 22). These factors might limit the direct translatability of our results to other tumor types and anatomical regions. However, our results indicate that DL-based tumor segmentation in MRI might not inherently require large datasets to be trained successfully. Our data-size ablation demonstrates the feasibility of investigating DL-based segmentation in settings with limited dataset sizes, providing a promising first step extending such research to rare pediatric tumors, where small datasets are unavoidable.

Our study used a standard nnU-Net approach for segmentation in a small dataset. We did not investigate other models or approaches specifically aimed at improving segmentation performance in low-data settings. One such strategy is the use of augmented data. Introducing small variations to the included scans, such as adding noise or rotational transformations, or exposing the model to more underrepresented cases, could potentially improve model robustness and performance. Although nnU-Net automatically applies data augmentation in its preprocessing steps, we did not specifically investigate the additional effect of dataset augmentation on model performance. Other approaches include using advanced training strategies or architectures, such as transfer learning and weakly supervised learning (23). Nevertheless, the choice of nnU-net was deliberate, as it is an established framework that can be used “out-of-the-box” with limited technical expertise, facilitating its implementation in centers with limited experience in deep learning.

Other limitations include the absence of statistical validation of our results, as this was limited by our small overall dataset size and nested training set-up. Furthermore, we only included the Dice score, while evaluation parameters like the Hausdorff distance might provide additional information. Next, we performed no lesion level segmentation in patients with a bilateral tumor, although accurate segmentation of both lesions is important during clinical application of the method. Additionally, our ground truth segmentations, although created following a protocol and reviewed by an experienced pediatric radiologist, might have inconsistencies influencing the results. This might particularly affect small tumors, causing a possible underestimation of segmentation performance. Lastly, this was a single-center study so independent validation of these results in centers with different scanning protocols is needed.

## Data Availability

The data supporting the conclusions of this article are not publicly available due to privacy and ethical restrictions. The data were collected under approvals and consent conditions that do not permit public sharing of the raw data.
